# Polyacrylate-magnetite nanocomposite as a potential multifunctional additive for lube oil

**DOI:** 10.1038/s41598-020-76246-4

**Published:** 2020-11-05

**Authors:** Koushik Dey, Gobinda Karmakar, Mahua Upadhyay, Pranab Ghosh

**Affiliations:** Natural Product and Polymer Chemistry Laboratory, Department of Chemistry, University of North Bengal, Darjeeling, 734013 India

**Keywords:** Materials chemistry, Engineering

## Abstract

The application of polymer nanocomposites (PNCs) in lubricant industry has attracted considerable interest due to their much enhanced properties compared to neat polymers. In this study, magnetite (Fe_3_O_4_) nanoparticles (NPs) were synthesized. Then PNCs were prepared by reinforcing these NPs in the homopolymer of dodecyl acrylate in different percentages. The characterization of the prepared NPs and PNCs was done by different analytical techniques. Thermal stability is determined through thermogravimetric analysis (TGA). Performance evaluation of the PNCs as viscosity index improver, pour point depressant and antiwear additive was carried out by blending them with a mineral base stock at different percentage ratios. Standard ASTM methods are followed to carry out the evaluations. It is found that with increasing the percentage of nanocomposites in the base stock, the overall performance of the furnished lubricant is enhanced.

## Introduction

Lubricants are the widely utilized material in automotive industry. Lubricants form a protective layer on the surface of machinery parts and thus reduce friction during turbo-chemical process. Except that, it improves the efficiency of engines, prolong their lifetime as well as economize energy. In the last few decades, use of oil-soluble additives in lubricant as effective friction reducer and anti-wear have been extensively studied in lubrication engineering^1–3^. But, the use of these additives increases pollution, toxicity, waste disposal in the environment. The application of nano materials as additive mitigates these limitations and opens new horizon in lubricant industry^4–6^. Nanoparticles were incorporated into lubricating oils to improve their tribological properties. In recent years, a large number of studies have been carried out to measure the potentiality of a range of inorganic nanoparticles as friction and wear reducer for lubricating oil^7–10^. Owing to excellent tribological and environmentally benign property, the nanoparticles have been preferred as an exceptional candidate in replace of traditional lubricating oil additives, particularly at higher load, higher sliding speed and higher frictional conditions^11–13^. The NPs adhere to the friction surfaces, resulting in the modification of the friction surfaces and the improvement of the tribological properties. Use of graphite nanosheets as lubricating oil additives improves tribological properties of paraffin oil^14^. The anti-wear property of paraffin oil was significantly improved by the incorporation of MoS_2_ nanoparticles^15,16^. Oxide based nanoparticles such as CuO nanoparticles exhibit good anti-wear and friction reduction properties^17^. Cai-Xiang et al.^18^ reported the anti-wear and friction reducing properties of CeO_2_ and TiO_2_ nanoparticles in lubricating oil. All the nanoparticles mentioned above are non-magnetic. The use of non-magnetic compounds containing elements like sulfur, phosphorus, lead, etc. as anti-frictional additive for lubricants also has some adverse effect on the environment. Magnetic NPs, on the other hand, is environmentally friendly and therefore has attracted considerable attention in this application area. Another advantage of using magnetic NPs as lubricant additive is its magnetic effect which originates in its remanent magnetization^19^. The lubricant with Fe_3_O_4_ NP additives forms a protective film adhering on the friction steel pairs and filled the gaps and cracks of the surface due to possible magnetic interaction between the lubricant and friction surface and also tribo-chemical reactions on the metal surface^20,21^. This results in significant improvement of antiwear properties of the lubricating oil.

Huang et al.^22^ reported the effect of magnetite nanoparticle on tribological property of paraffin oil which showed improvement of load carrying capacity and anti-wear property of the formulated lubricant compared to pure paraffin oil. Ziang et al.^23^ described the tribological and tribochemical properties of magnetite (Fe_3_O_4_) nanoflakes as additive in mineral base fluids. From the above discussion it is revealed that magnetite nanoparticle and other oxide based nanoparticles are used only as anti-wear and friction reducing additives in lubricating oil. Furthermore, acrylate based polymers are known to perform as good viscosity index improver (VII)^24–26^, pour point depressant (PPD)^27^. They were not known to act as good anti-wear and friction reducing additives. Hence to impose multifunctional character (VII, PPD, anti-wear, friction reducing) into the lubricant additives, the authors planned to prepare PNCs of dodecylacrylate by using magnetite (Fe_3_O_4_) NPs as fillers.

## Experimental section

### Materials

Acrylic acid (99%), dodecyl alcohol (98%) and hydroquinone (> 99%) were purchased from Sigma-Aldrich, India. Methanol (98%, Thomas Baker Pvt. Ltd.) and hexane (99.5%, S.D. Fine-Chem Limited) were used after distillation. Conc. H_2_SO_4_ (98%, Merck Specialties Pvt. Ltd.) was used as received. AIBN (GC 98%), obtained from Spectrochem Pvt. Ltd. Mumbai (India) was recrystallized from CHCl_3_-MeOH before use. Iron(II) sulphate heptahydrate (> 99%), potassium nitrate and potassium hydroxide (99.9%) were collected from Merck Specialties Pvt. Ltd. The base oil, property of which is mentioned in supplementary section, was collected from IOCL, Kolkata, India.

### Preparation of dodecyl acrylate

Dodecyl acrylate (DDA) was prepared by esterification of acrylic acid with dodecyl alcohol in 1.1:1 mol ratio. The reaction was performed in a resin kettle in presence of concentrated sulfuric acid as a catalyst, 0.25% hydroquinone with respect to the reactants as polymerization inhibitor, and toluene as a solvent. The reaction was carried out under nitrogen atmosphere. The reaction mixture was heated gradually from room temperature to 403 K using a well-controlled thermostat. The extent of reaction was followed by monitoring the amount of water liberated during reaction. After completion of the reaction, the ester dodecyl acrylate (DDA) was collected.

### Purification of the prepared ester (DDA)

To purify the product, a desired amount of charcoal was added to the ester, followed by reflux for 3 h and then filtered. The filtrate was washed repetitively with 0.5 N sodium hydroxide solution to ensure complete removal of unreacted acid. To remove traces of sodium hydroxide, purified ester was washed several times with distilled water. The ester was then left on calcium chloride overnight and recollected by distillation under reduced pressure. This purified ester was then used in the polymerization process.

### Synthesis of homo polymer of DDA

The polymerization was carried out in a four-necked round bottom flask fitted with a condenser, stirrer, thermometer and an inlet for the nitrogen insertion. Required amounts of dodecyl acrylate and initiator (AIBN, 0.5% w/w) were taken in the flask and toluene was also added as solvent. The reaction temperature was controlled at 353 K for 6 h. Then the reaction mixture was poured into methanol solvent with stirring to cease the polymerization and a precipitate was appeared. The precipitated polydodecyl acrylate (A), PDDA, was further purified by frequent precipitation of its hexane solution with methanol followed by drying under vacuum at 313 K.

### Preparation of magnetite (Fe_3_O_4_) nanoparticle

Magnetite nanoparticles were synthesized following the method reported by Bruce et al.^28^ Solutions of iron(II) sulphate heptahydrate (1.67 g, 6 × 10^–3^ mol) in 50 ml deionized water, potassium nitrate (1.01 g, 1 × 10^–2^ mol) in 10 ml of deionized water and 2.5 M potassium hydroxide solution were prepared. 1% (w/w) of surfactant (CTAB) was mixed with the iron salt solution under vigorous stirring for 2 h. Solution of potassium nitrate was added to this solution and stirred for another half an hour. Then 10 ml of 2.5 M potassium hydroxide (2.5 × 10^–2^ mol) was slowly added to the above solution. The reaction mixture was heated to 100 °C under nitrogen atmosphere and maintained at this temperature for 2 h. The nitrogen flow was then turned off and the mixture was cooled down to room temperature. After cooling, the black precipitate was repeatedly washed with deionized water, centrifuged and allowed to dry under vacuum at 323 K overnight^29^.

### Preparation of poly dodecyl acrylate-Fe_3_O_4_ nanocomposites

PDDA-Fe_3_O_4_ nanocomposites were prepared by blending the PDDA/toluene solution and nano- Fe_3_O_4_ particles. The PDDA-Fe_3_O_4_ suspension was prepared as follows: 5 g of PDDA were dissolved in toluene and the required amount (0.5, 1 and 1.5 mg) of nano-Fe_3_O_4_ particles were added to it under ultrasonic wave with vigorous stirring. The suspension was then poured into a glass plate and allowed the tolune to evaporate naturally and a semi solid mass of polymer-nanocomposites was obtained. It was then characterized. The relative compositions of different composites with their designations are mentioned in Table 1.

**Table 1:** Designation and composition of poly dodecylacrylate-nano magnetite composites.

Designation	Composition	
	Polymer (g)	% of nano Fe_3_O_4_
A	5	0
F-1	5	0.01
F-2	5	0.02
F-3	5	0.03

## Measurements

### Spectroscopic measurements

The spectral characterization of the polymers and the composites was performed by FTIR and NMR techniques. IR spectra were recorded on a Shimadzu FT-IR 8300 spectrometer using 0.1 mm KBr cells at room temperature within the wave number range of 400–4000 cm^−1^. NMR spectra were recorded in Bruker Advance NEO 400 MHz FT-NMR spectrometer using 5 mm BBO probe and CDCl_3_ solvent. TMS was used as reference material.

### Thermo gravimetric analysis (TGA)

The thermal stabilities of the prepared homo polymer and polymer nanocomposite were determined by a thermo gravimetric analyzer (Shimadzu TGA-50) using an alumina crucible in air. The system was run at a heating rate of 10 °C/min. The percentage of weight loss (PWL) of the samples with rise in temperature was calculated.

### Determination of average molecular weights of PDDA

The number average molecular weight (M_n_) and weight average molecular weight (M_w_) of PDDA were determined by SEC-GPC. The polydispersity index was also calculated. In this method THF (0.4%, w/v) of HPLC grade was used as mobile phase in the Waters 2414 GPC system (polystyrene calibration) at 35 °C. Sample solutions (0.4% w/v in THF) are prepared by dissolving ~ 4 mg of polymer per ml THF and filtering (0.45-μm Millipore PTFE) to remove suspended particulates. The pump flow rate is 1.0 ml/min with THF as the carrier solvent, and injection volumes are set to 20 μL. The polydispersity index, which indicates the nature of the distribution of the molecular weights in the polymers, was also calculated. The data of this study is mentioned in supplementary material.

### Characterization of nano-Fe_3_O_4_ and the composites by XRD and different electron microscopes (SEM/TEM)

The synthesized Fe_3_O_4_-nanoparticles and the composites were characterized by Field Emission Scanning Electron Microscope (FE-SEM, INSPECT F50, FEI), Transmission Electron Microscope (TEM) and X-ray diffraction (XRD, Advance D8, Bruker).

### Magnetic characterization of the NPs

The magnetic characterization of the NPs was done using a Vibrating Sample Magnetometer (VSM) at 300 K with a magnetic field − 10 kOe to + 10 kOe.

### Determination of viscosity index

Viscosity index (VI) of the polymeric additives was determined in the paraffinic base oil to evaluate the efficiency of the prepared polymeric additives as viscosity modifier (VM). The viscosity index (VI) of the base oil blended with additives at different concentration levels was evaluated according to the ASTMD2270 method using the equations as reported by Tanveer and Prasad^30^.

### Determination of pour point

The pour point depressant property of base oils blended with the polymeric additives was determined by measuring pour points of the lubricants on a Cloud and Pour Point Tester model WIL-471(India) according to ASTM D97 method. In this case also five different concentrations of the additives were used for each sample.

### Evaluation of tribological properties

The anti-wear and friction modifier performance of the lubricant compositions were evaluated in terms of wear scar diameter (WSD) by Four-ball wear test apparatus (FBWT) following ASTM D 4172–94 method^31^. In this experiment, 392 N (40 kg) load at 75 °C for 30 min. was employed to measure the wear scar diameter (WSD). The diameter and rotating speed of the ball were 12.7 mm and 1200 rpm respectively. The details procedure is described in our previous publication^32^.

## Results and discussion

**Figure 1:**
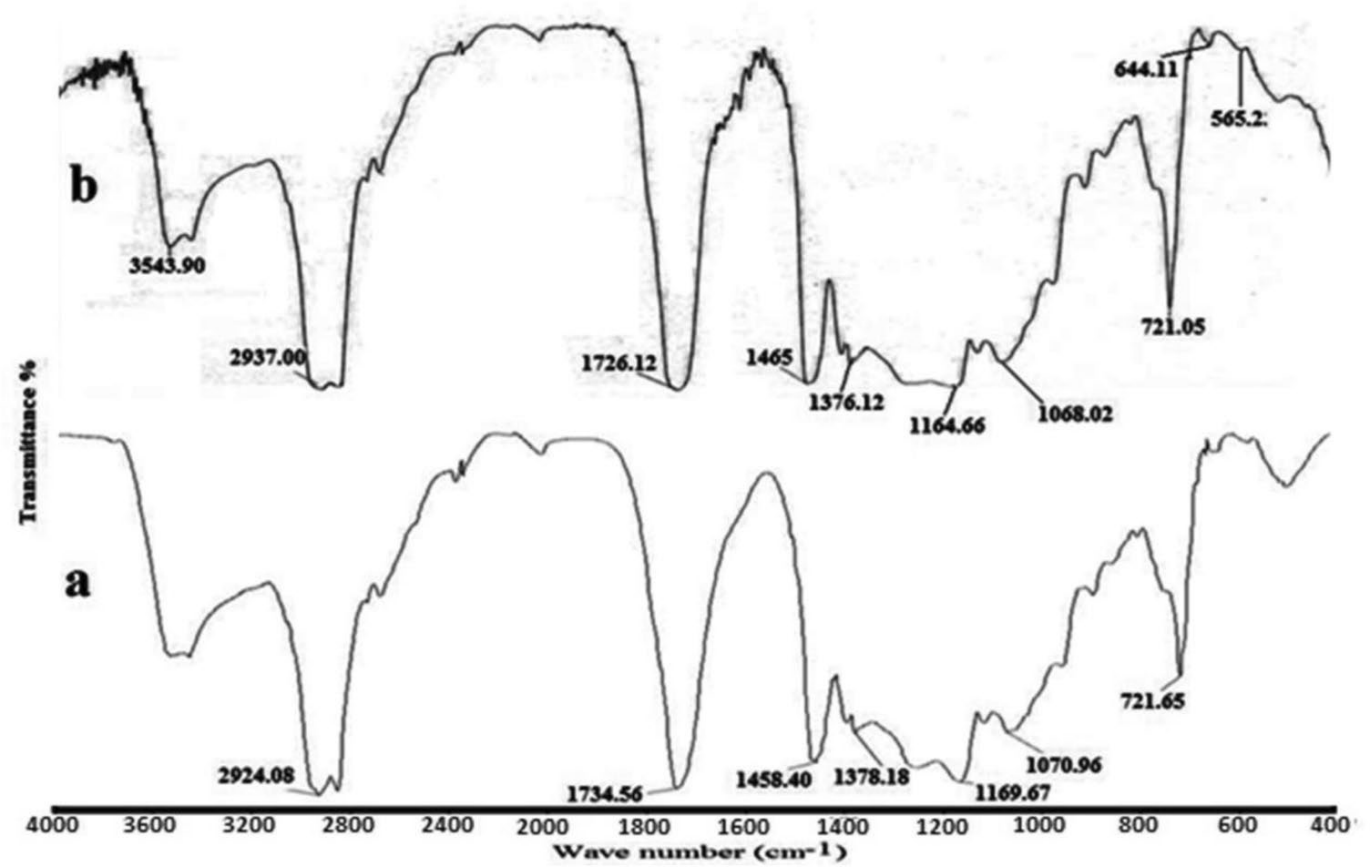
FT-IR spectra of (**a**) polymer (A) and (**b**) polymer/Fe_3_O_4_ nanocomposite (F-3).

The FT-IR spectrum of homopolymer of dodecyl acrylate (A) and one of the PNCs (polymer composite F-3) is shown in Fig. 1. The absorption band at 1734.56 cm^-1^ in Fig. 1a indicated the ester carbonyl stretching vibration of polymer A. The Fig. 1b of polymer/Fe_3_O_4_ nanocomposite showed the absorption for ester carbonyl group at 1726.12 cm^-1^. This shifting of carbonyl stretching frequency may be due to some association of nanomagnetite and poly dodecyl acrylate. Peaks at 1465, 1458.40, 1376.12 and 1378.18 are for asym and sym bending vibrations of C–H bonds of –CH_3_ and –CH_2_– groups of F-3 and A. Peaks in the range 1164.66, 1169.67, 1068.02, 1070.96 cm^−1^ are for C–O stretching vibration of carboxylate ester group. Peaks at about 721 cm^−1^ are for C–H bending vibration of the paraffinic chain. Broad peaks in the range 2924.08–2937 cm^−1^ are for stretching vibration of paraffinic C–H bonds of –CH_2_– groups. There is no significant peak observed in the range of olefinic bonds which supported the formation of the polymer.

**Figure 2:**
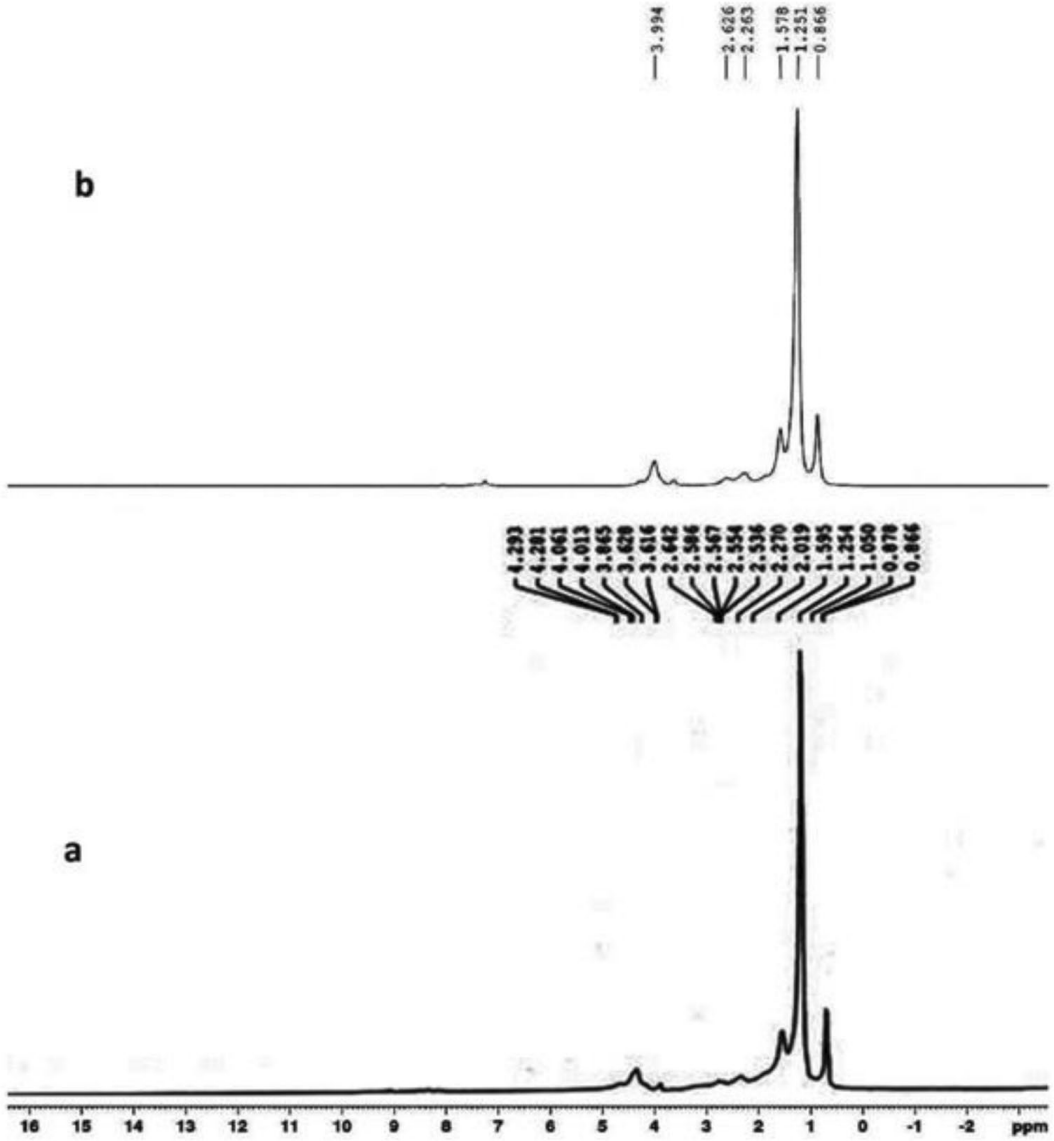
^1^H NMR spectra of polymer (A) and the PNC (F-3).

**Figure 3:**
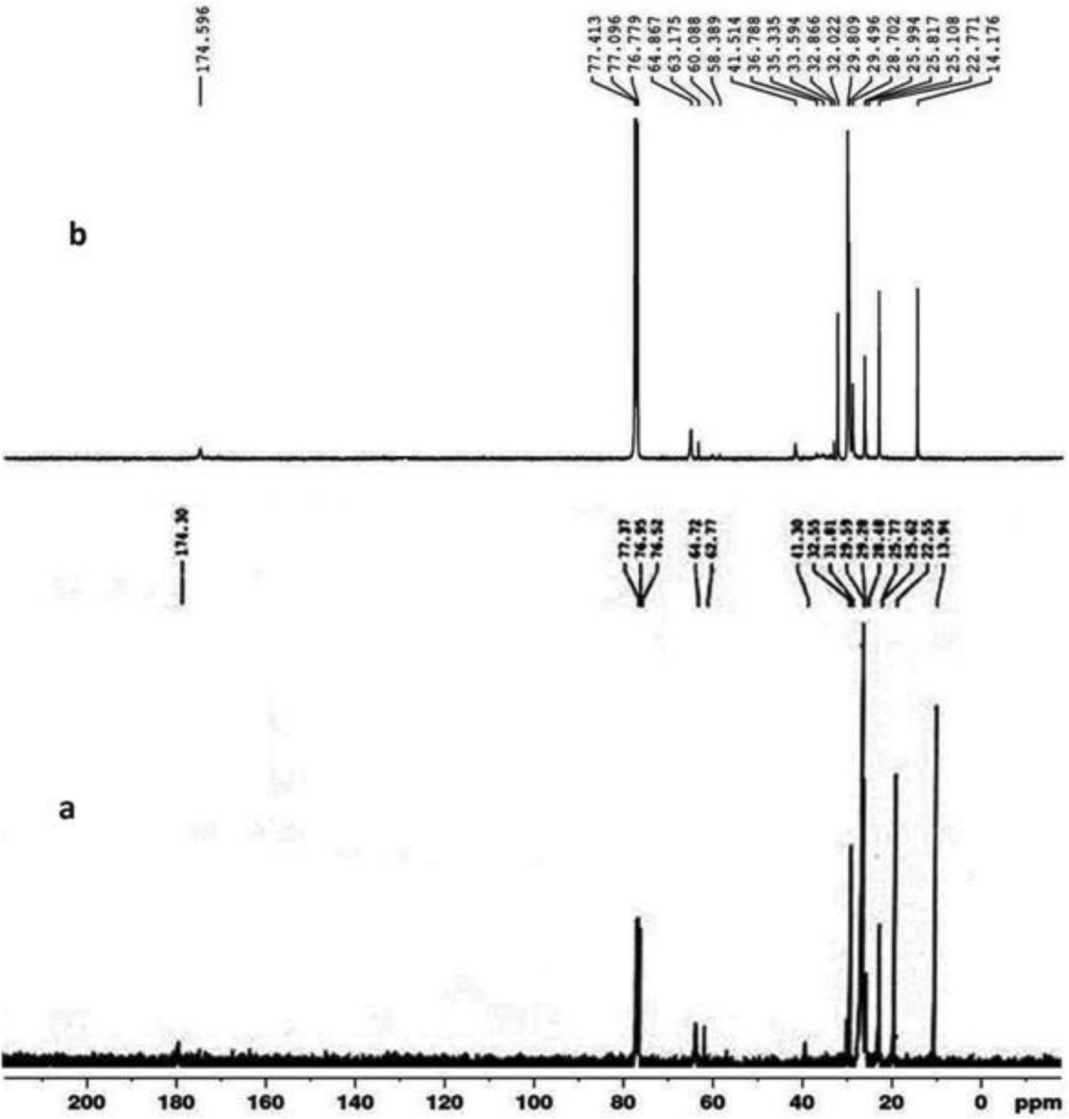
^13^C NMR spectra of polymer (A) and the composite (F-3).

The ^1^HNMR spectra of PDDA and the polymer nanocomposite (F-3) are shown in Fig. 2. In Fig. 2, (a) represents the spectrum of PDDA and (b) for the composite. Abroad singlet centered on 4.013–4.293 ppm in (a) is due to the protons of –OCH_2_ group of PDDA. Methyl protons of dodecyl chain appeared between 0.866 to 0.878 ppm. The absence of singlet between 5 and 6 ppm indicated the absence of any vinylic protons in the polymer. The ^1^HNMR of the polymer nanocomposite, (b), showed a peak at 3.99 ppm which is due to the protons of –OCH_2_ group of the acrylate polymer. Methyl and methylene protons appeared in the range 0.866–1.578 ppm. Peaks appeared in the range 2.263 ppm are due to protons of α carbon to the carbonyl group of the ester. The ^13^C spectra of PDDA and the PNC (F-3) are depicted by (a) and (b) respectively in Fig. 3. In the ^13^C NMR spectrum of PDDA, (a), the carbonyl carbon appeared at 174.3 ppm along with other SP^3^ carbons appeared in the range of 64.72–13.94 ppm. The carbonyl carbons of the composite (F-3) appeared at 174.59 ppm as shown in (b). Peaks appeared from 14.176 to 41.514 ppm are for the sp^3^ carbons of alkyl chains of PNC and 58.39–77.413 ppm are for the carbons of ester groups. The intensity of NMR signals of the polymer composites are appeared poor which may be due to interference of microwave frequencies with the magnetic material presence in the composite.

**Figure 4:**
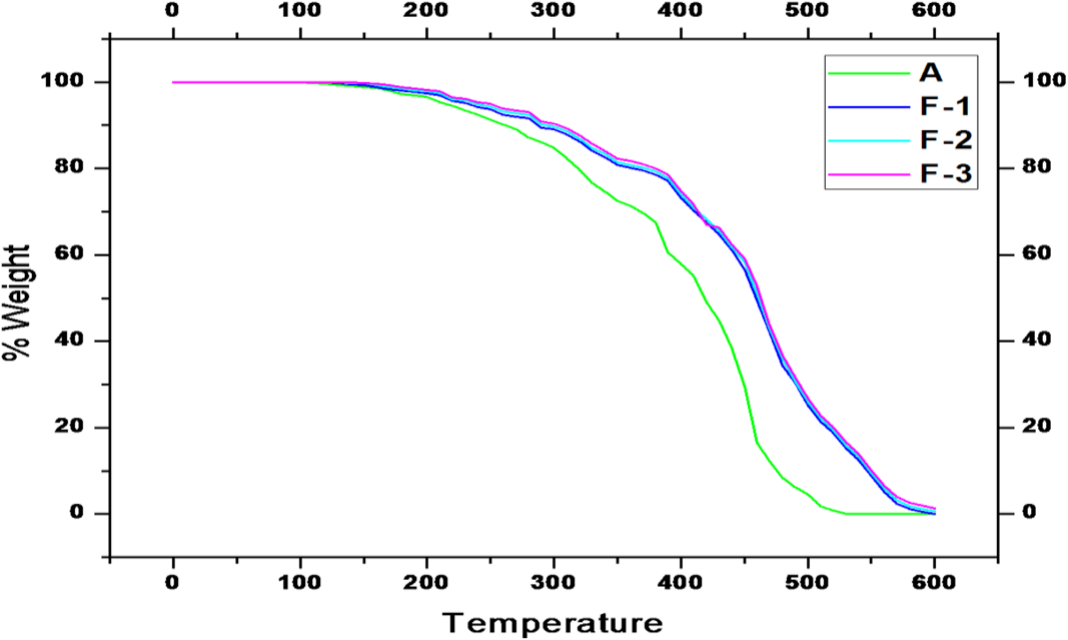
TGA data of polymer (A) and polymer/Fe_3_O_4_ nanocomposite (F-1, F-2 and F-3).

The TGA data of the polymer (A) and polymer/Fe_3_O_4_ nanocomposites (F-1, F-2 and F-3) are depicted in Fig. 4. It was found that, the incorporation of nano-Fe_3_O_4_ into A increases the thermal stability of the composites. At 380 °C, the percentage of degradation of A, F-1, F-2 and F-3 were 32.42%, 21.35%, 20.64% and 20.01% respectively, whereas at 490 °C, the percent of weight loss of A, F-1, F-2 and F-3 were 93.71%, 69.56%, 69.12% and 68.12% respectively. The degradation of the polymer was inhibited by the addition of nano-Fe_3_O_4_ and as a result the polymer composites showed improved thermal stabilities. The decrease of mobility of polymer chain and the tendency of magnetite nanoparticle to eliminate free radicals may be the key effects accountable for this enhancements^33^.

**Figure 5:**
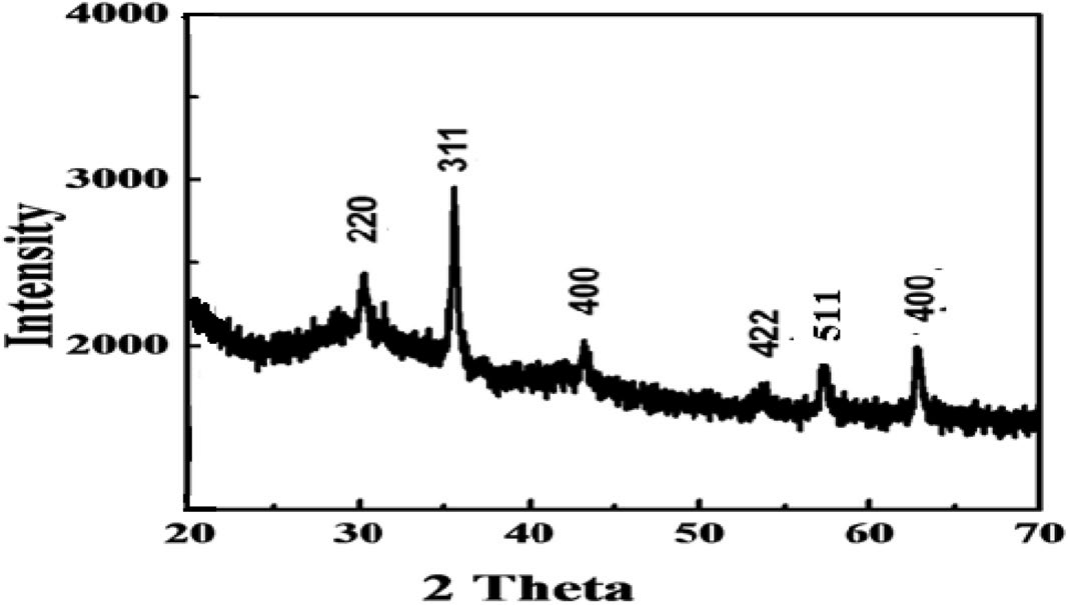
XRD pattern of prepared nano-Fe_3_O_4_.

Figure 5 showed the X-ray diffraction pattern of the prepared magnetite nanoparticle. It was taken within the range of 20°–70° (2θ). The six most intense peaks at 30.3°, 35.6°, 43.2°, 53.6°, 57.1° and 62.8° respectively were markedly observed and was found very similar as obtained for magnetite nanoparticles elsewhere^29^. The purity of magnetite nanoparticles was confirmed by the absence of peaks due to other forms of iron oxides like maghemite or hematite in the sample. Hematite nanoparticles shows nine intense peaks in the diffraction angle from 6° to 70°^34^.

**Figure 6:**
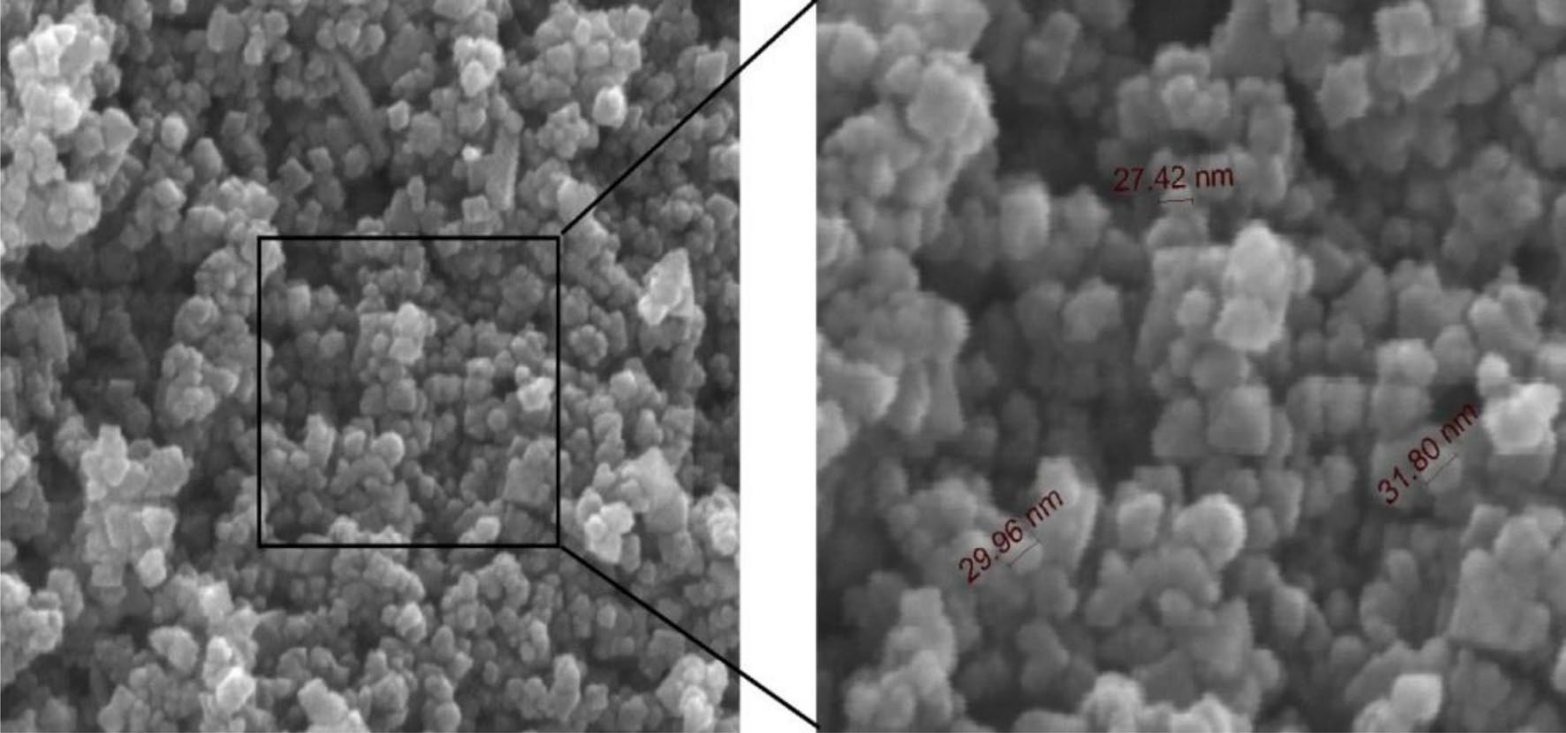
SEM images of prepared nano-Fe_3_O_4_ at different magnifications.

**Figure 7:**
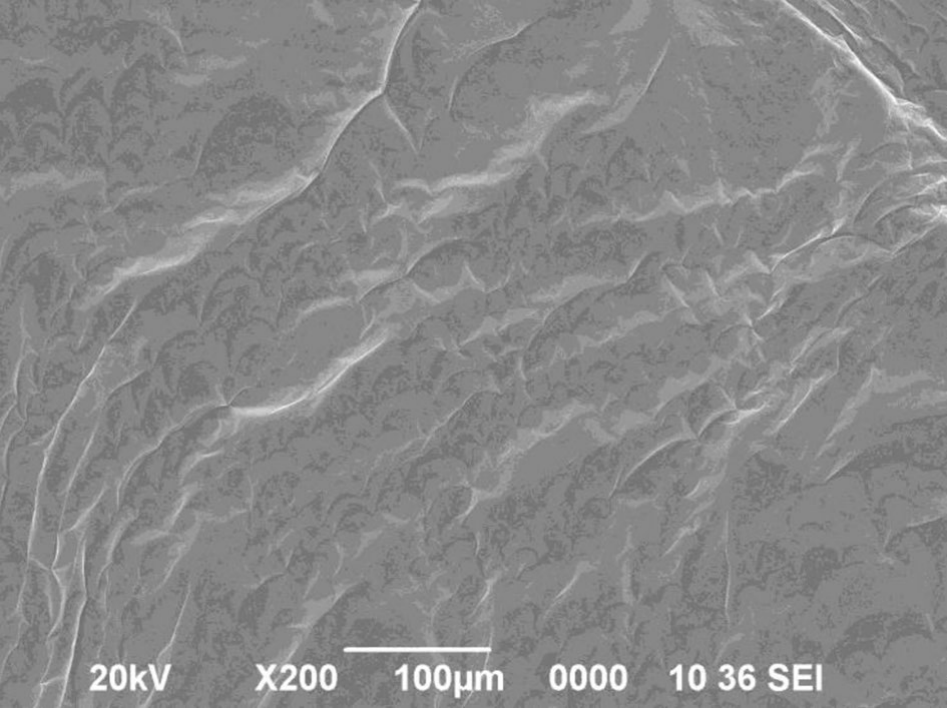
SEM images of prepared Polymer/nano-Fe_3_O_4_ (F-3) composite.

Figures 6 and 7 showed the scanning electron micrograph of the prepared magnetite nanoparticle and the composite respectively. Shapes of the particles marked in Fig. 6 showed that the particles were nearly spherical. It can be seen from the figure that the particles have an average size of about 29 ± 2 nm. The formation of the nanoparicles is further confirmed by TEM image mentioned in the supplementary materials.

Magnetic behavior of these nanoparticles depends on temperature and particle size. The magnetic characterization was done using a vibrating sample magnetometer (VSM) at 300 K with a magnetic field − 10 kOe to + 10 kOe^35^. The symmetric hysteresis loop indicated a superparamagnetic behaviour of the nano particles in the applied field with zero coercivity and remanance values. The saturation magnetization (M_s_) of the magnetic nanoparticles is 74.23 emu/g. The saturation magnetization value is related with the size of the NPs. It increases with increasing size of the NPs. The higher value of Ms in this study give evidence about the average size of NPs as obtained by SEM study. The diagram of the magnetic behavior of the nanoparticles as obtained by the experiment was mentioned in the supplementary material.

**Figure 8:**
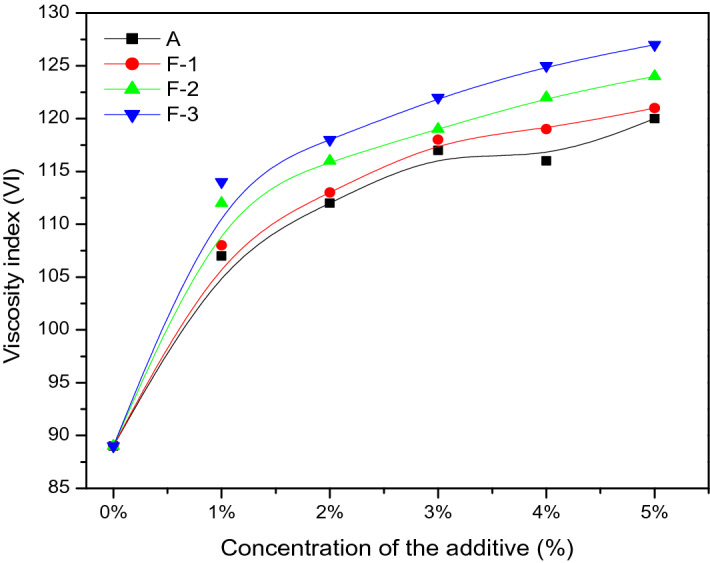
Plot of viscosity index of the lube oil blended with additives at different concentration levels.

Figure 8 represents the viscosity index values of the lube oil blended with additives of different concentrations. It is observed that, the viscosity index (VI) values of the base oil blended with the polymer/nano-Fe_3_O_4_ composites (F-1, F-2 and F-3) are better than the pure polymer (A) at every concentration. For both type of additives, there is always a steady increase of VI values with the increase in additive concentration in base stock. The lubricant blended with 5% F-3 additive showed highest increment (42.7%) in viscosity index compared to pure base oil. This is due to increase in total volume of polymer micelles in lubricant with increase of concentration of the additives. The nanoparticles separate the polymer chains in the matrix which is responsible for increase of the volume also. The greater volume of composite units in solution contributes higher VI of the lubricant.

**Figure 9:**
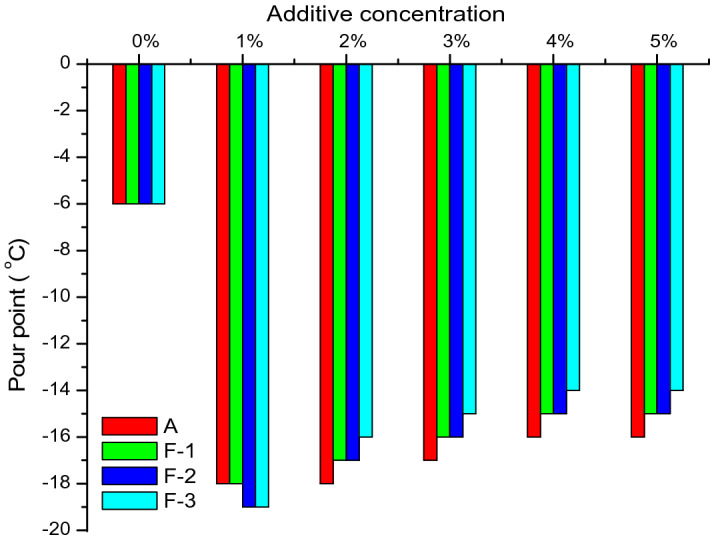
Plot of pour points of the lube oil blended with additives at different concentration levels.

The PPD of lubricant compositions at different percentage of additives varying from 1 to 5 wt% was tested and the results are depicted in Fig. 9. The results showed that the additives (A, F-1, F-2 and F-3) are efficient as PPD and the efficiency decreases with increasing the concentration of additives. The PPD of all the lubricants blended with composites is very similar with that of pure polymer. That means incorporation of nanoparticles into the polymer matrix does not affect the PPD property compared to neat polymer (A).

**Figure 10:**
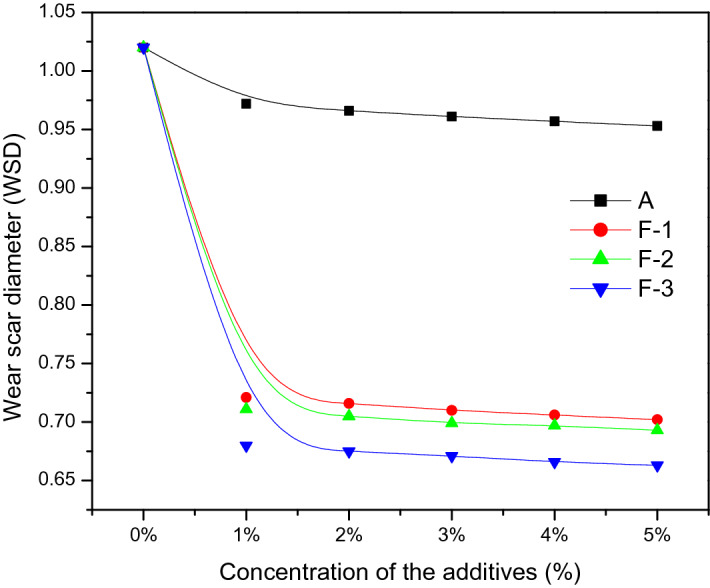
Wear scar diameter (WSD) of the lube oil blended with additives at different percentage (w/w).

The tribological properties of all the lubricant compositions (A, F-1, F-2 and F-3) were tested through measuring WSD by FBWT apparatus employing 40 kg load and the results were depicted in Fig. 10. The AW performance of the base oil is significantly enhanced when the additives are blended with it and is indicated by the gradual decrease in WSD values with increasing the percentage of the additives. The nano-iron particles in lubricant composition interact with the metal surface during tribochemical process which decreases wear^36^. The lubricant containing 5% nano-additive (F-3) exhibited lowest WSD (35% decrease) compared to pure base oil.

## Conclusion

In this study nano-Fe_3_O_4_ was synthesized and used as filler particles in the formation of polydodecyl acrylate composites. The characterizations of both the polymer and composites were studied by SEM, TEM, XRD and spectral analysis techniques (FTIR, NMR). Through TGA data we showed that thermal stability of polymer was improved significantly by the incorporation of nanomagnetite into the polymer matrix. During their evaluation as additive for lubricant, it was found that all the nano-blended composites showed improved performance as viscosity modifier and anti-wear additives for lube oil. Therefore, the above study is definitely a potential approach to design multifunctional additives for lubricating oil.

## Supplementary information

Supplementary Information.
