# Supplementary material for: Polyacrylate-magnetite nanocomposite as a potential multifunctional additive for lube oil
Source: Sci Rep. 2020 Nov 5;10:19151. doi: 10.1038/s41598-020-76246-4 (PMC7644704; doi:10.1038/s41598-020-76246-4)
Supplement: Supplementary file 1 — Supplementary Information. [file 41598_2020_76246_MOESM1_ESM.docx]

**Polyacrylate-magnetite nanocomposite as a potential multifunctional additive for lube oil**

**Koushik Dey, Gobinda Karmakar, Mahua Upadhyay and Pranab Ghosh***

Natural Product and Polymer Chemistry Laboratory, Department of Chemistry,

University of North Bengal, Darjeeling, 734013, India

E-mail: [pizy12@yahoo.com](mailto:pizy12@yahoo.com). Tel.: +91 9474441468. Fax: +91 353 2699 001

**Supplementary Material**

Contents

- Physical properties of base oil………………………………………..Table S1
- Average molecular weights of PDDA ………………………….……Table S2
- TEM image of magnetite nanoparticle.…………...............................Figure S1
- Magnetization curve of magnetite NPs obtained by vibrating sample magnetometer (VSM) at 300 K...........................................………………………....Figure S2

| **Table S1. Physical properties of the base oil** | |
| --- | --- |
| Properties | Value |
| Density (kg.m^-3^) at 313K | 918.68 |
| Viscosity (cSt) at 313K | 20.31×10^-6^ |
| Viscosity (cSt) at 373K | 3.25×10^-6^ |
| Viscosity index | 89.02 |
| Cloud point (°C) | -8 |
| Pour point (°C) | -6 |

cSt: centistoke

**Table S2. Average molecular weights of PDDA**

| Polymer sample | M_n_ | M_w_ | PDI |
| --- | --- | --- | --- |
| HOMO POLY DODECYL ACRYLATE | 33789 | 38412 | 1.14 |
|  |  |  |  |


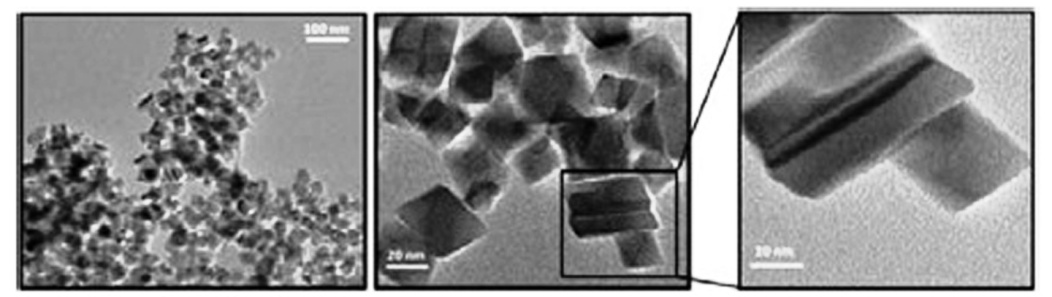


Figure S1: TEM image of magnetite nanoparticles


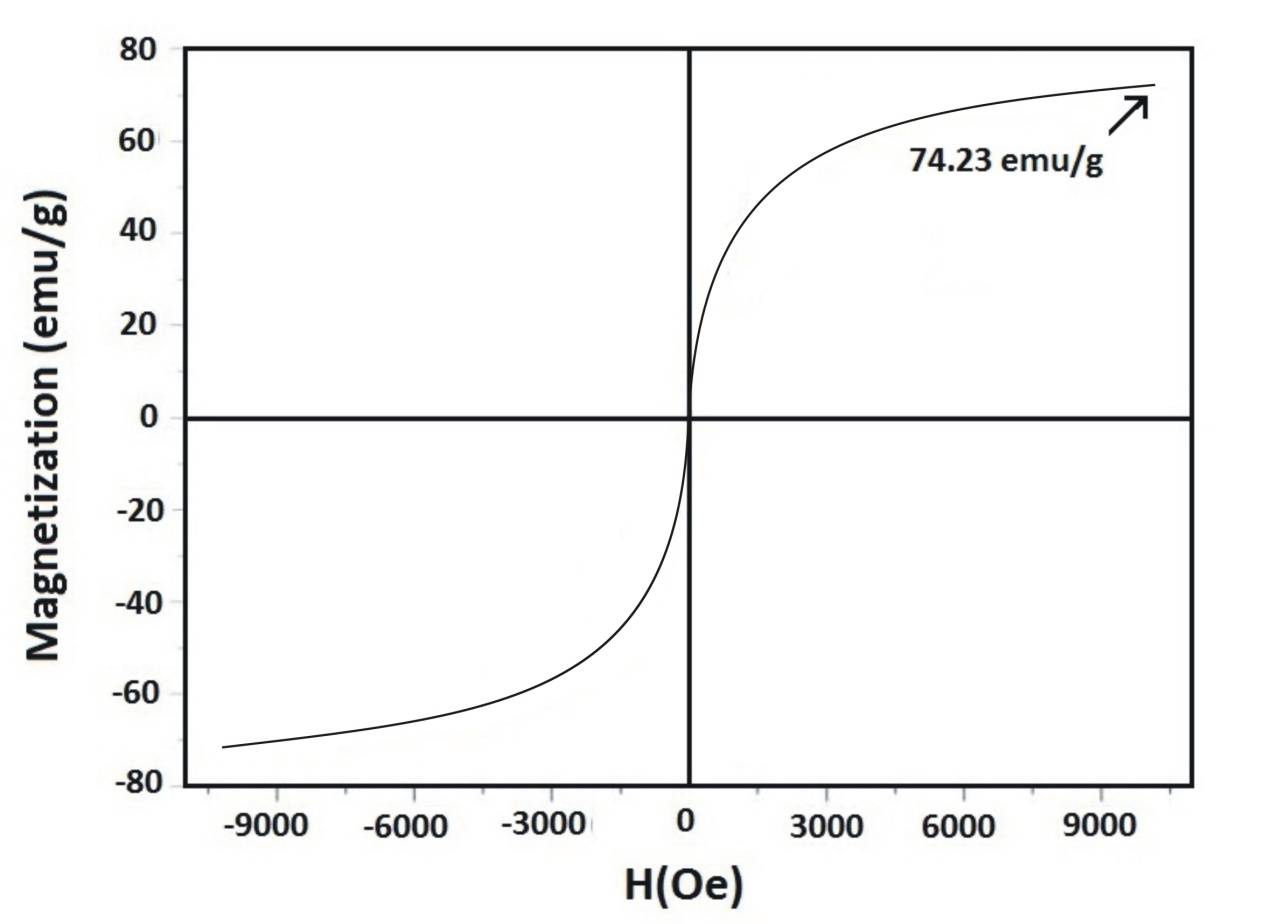


**Figure S2: Magnetization curve of magnetite NPs obtained by vibrating sample magnetometer (VSM) at 300 K**
